# Eastern redcedar roots create legacy effects that suppresses growth of prairie species

**DOI:** 10.1002/ece3.10775

**Published:** 2023-12-11

**Authors:** Leland D. Bennion, David Ward

**Affiliations:** ^1^ Department of Biological Sciences Boise State University Boise Idaho USA; ^2^ Department of Biological Sciences Kent State University Kent Ohio USA

**Keywords:** allelopathy, eastern redcedar, grasslands, *Juniperus virginiana*, legacy effects, range expansion, woody encroachment

## Abstract

The expansion of woody species from their historical ranges into grasslands is a global problem. Understanding the mechanisms that enable species to successfully establish and then re‐encroach following their removal is critical to effectively managing problem species. Legacy effects are a mechanism that could be critical to the reestablishment of woody encroachers following their removal. Legacy effects occur when a species alters the biotic and abiotic environment in a way that affects communities that establish subsequently. In this study, we assess whether the eastern redcedar (*Juniperus virginiana*), a North American woody encroacher, generates legacy effects that affect communities that establish following removal of this species from an experimental grass community. We conducted a series of experiments to evaluate the effects of *J. virginiana*, roots on the germination and growth of grasses and to determine if the effects of root‐addition treatments were derived from a microbial or allelopathic origin. Aqueous extracts of *J. virginiana* roots were found to inhibit the germination of grasses. We found escalating suppression of overall community biomass and the biomass of each individual species with increasing root treatments. Finally, we determined the origin of the observed suppressive effect is unlikely to be of microbial origin. *Synthesis*: Our results suggest that *J. virginiana* exudes an allelochemical into soils that inhibits the growth of certain grasses and thus has the potential to have legacy effects on future occupants. We suggest that the inhibition of the development of grasses in areas where *J. virginiana* has been removed is a mechanism that may favor the reestablishment of *J. virginiana*. Our results indicate the legacy effects of *J. virginiana* must be considered when conducting removal and restoration of *J. virginiana* infested lands.

## Graphical abstract

The root materials of eastern redcedar (*Juniperus virginiana*) inhibit the growth and germination of common prairie species. These legacy effects could promote reestablishment of the encroaching redcedar following removal.

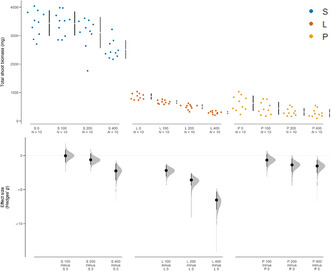

## 1 INTRODUCTION

Species are moving into habitats outside their historical range in response to human‐mediated translocation, habitat change, and alteration of abiotic conditions (Hoegh‐Guldberg et al., 2008; Poland et al., 2021). The introduction of species to a new range is deemed problematic when it results in negative changes to the ecosystem, habitat, or preexisting resident species (e.g. Lambertini et al., 2011; Simberloff, 2011). These species can be of non‐native or native origin and are frequently referred to as invasive species or range expanders, respectively (Tomiolo & Ward, 2018). The introduction of a novel species into a community can lead to changes in competitive interactions, resource availability, and soil microbial communities (Callaway & Ridenour, 2004; Kourtev et al., 2002; Perkins & Hatfield, 2014). Due to the actual or potential negative effects on systems in their new range, novel species are frequently the target of management that seeks to extirpate them from an area (Pearson et al., 2016). However, there is some potential for the effects of novel species to persist following their extirpation and thus influence future residents, a phenomenon known as the legacy effect (Perry, 2008).

In ecology, legacy effects can describe the long‐term indirect effects of a species following their removal from an area. These effects can alter the successional trajectory of a system and are often attributed to human‐mediated disturbance, herbivory, and invasive species (Åkesson et al., 2021; Cuddington, 2011; Kostenko et al., 2012). Invasive species can alter the successional trajectory and community composition of future occupants through modifications to the soil environment and the resident soil‐microbial community (Davis et al., 2005; Jordan et al., 2012). These legacy effects are often microbially‐mediated, although abiotic alterations to the rhizosphere such as allelopathy can also be important (Bever, 2003; Del Fabbro & Prati, 2015; Kardol et al., 2007). Microbially mediated plant–soil feedback is a commonly studied mechanism wherein the impact of novel plants on their near‐soil environment alters the fitness of subsequent con‐ and heterospecifics (De Long et al., 2023; Levine et al., 2006; Suding et al., 2013). The impact of microbially mediated feedbacks on plant growth can be reinforced or moderated by abiotic conditions such as the physical and chemical characteristics of soils (Bennett & Klironomos, 2019; Ehrenfeld et al., 2005).

Allelochemicals are compounds released by plants that can adversely affect their neighbors directly or indirectly by interacting with soil microbiota (Inderjit & Callaway, 2003) and are widespread across plant clades (Kalisz et al., 2021). There are numerous examples of plants that have a direct impact on neighboring species through the production of toxic chemicals (Callaway & Ridenour, 2004; Dorning & Cipollini, 2006; Zhang et al., 2021). Chemicals produced by plants can indirectly affect neighbors by altering the soil microbial community and local nutrient cycles (Cipollini et al., 2012; Zhang et al., 2019). Many examples of adverse allelopathic effects by a plant on neighbors include both direct and indirect effects (Chen et al., 2019; Dommanget et al., 2014; Grove et al., 2012).

### 1.1 Woody encroachment

Woody encroachment by native and non‐native species is pervasive in North American grasslands (Barger et al., 2011; Morford et al., 2022). Human‐mediated disturbances such as land‐use changes, overgrazing, greatly extended fire regimes, and climate change (including elevated CO_2_) have created a positive feedback that favors conversion from grasslands to woody systems (Heisler et al., 2003; Miller et al., 2017; Ratajczak et al., 2014). One of the most important encroaching species in North America is the eastern redcedar (*Juniperus virginiana*), which is an aggressive range expander in natural and disturbed gaps in the eastern portion of its range but is also known for its dispersal in the Great Plains that is rapidly converting grasslands and prairies to woodlands (Wang et al., 2017).

The legacy effects of *J. virginiana* encroachment on prairie plant communities are understudied. Recent research indicates burned redcedar stands may allow herbaceous biomass to recover to levels that are similar to unencroached areas (Limb et al., 2014). However, the result is often short lived due to rapid reestablishment of *J. virginiana* (Bielski et al., 2021; Fogarty et al., 2021). There is evidence that *J. virginiana* may contain allelopathic compounds that suppress the growth of certain grass species and thus favor reestablishment of *J. virginiana* following removal (Bennion & Ward, 2022; Stipe & Bragg, 1989).

We conducted a series of experiments to determine if *J. virginiana* roots are a possible source of allelochemicals. In the first experiment we tested the effect of an aqueous extract of *J. virginiana* roots on the germination of four common prairie grass species. Next, we wanted to evaluate whether roots that were washed thoroughly retained enough microbes to cause a microbially‐mediated effect that could confound detection of allelopathy. We conducted a root‐sterilization experiment to test if there is an observable difference in the response of grass growth in roots that had been sterilized and those that were not. Finally, we established a greenhouse study that assessed the legacies of *J. virginiana* on a community of grasses following removal. We examined the strength and direction of legacy effects derived from *J. virginiana* roots on individual species and on the experimental plant community. We predicted that if *J. virginiana* roots contain potential allelochemicals, then:

(1) We would observe a reduction in the germination rate of one or more grasses with the addition of aqueous root extract.

(2) In the root‐sterilization experiment, we predicted that the control biomass would exceed all other treatments and that there would be no difference in biomass production among treatment groups.

In the community experiment, if the addition of *J. virginiana* roots introduced a source of allelochemicals, we hypothesized that we would observe a decrease (3) in the cumulative and (4) individual biomass of grasses with increasing levels of root addition of redcedars in each soil type.

(5) If there was a differential effect of *J. virginiana* roots on the growth of grass species, we hypothesized that we would observe changes in the proportion of biomass of each species relative to the overall biomass of all other species.

## 2 MATERIALS AND METHODS

Each of the three following experiments used *J. virginiana* roots as a treatment. Greenhouse‐reared seedlings were obtained from Pinelands Nursery, New Jersey. Seedlings were grown in Promix® potting soil for ~3 years prior to use in this experiment. The height of these trees ranged from ~0.5 to 1 m. At the time of harvest for all experiments, aboveground tissue was removed at the root collar and roots were thoroughly washed to remove as much soil as possible. Additional handling prior to use as a treatment is detailed in each experimental description.

### 2.1 Germination experiment

Following harvest as detailed above, *J. virginiana* roots were dried for 5 days in a 60°C oven. Distilled water was used as an extractant at a ratio of 10 mL water per gram of *J. virginiana* root material. The mixture was allowed to sit for 84 h prior to being decanted through Whatman No 1 filter paper. A portion of the extractant was further diluted to a concentration of 20 mL distilled water/g root material. Seeds of the common cool‐season C_3_ grasses *Pascopyrum smithii*, *Elymus canadensis*, and *Bromus inermis* and the warm‐season C_4_ grass *Bouteloua curtipendula* were selected for use in the germination experiment. Small petri dishes were lined with Whatman #2 filter paper. Ten seeds from each of the test species were added to each petri dish. The filter paper in each petri dish was moistened with one of three treatment solutions: the control (distilled water), 5% concentration treatment, and 10% concentration treatment. Seeds and filter paper were remoistened every other day. There were five replicates of each seed type and treatment combination. Petri dishes were monitored daily for 15 days. Seeds that germinated were removed from the petri dishes and the day of germination was noted. For each species, we conducted a one‐way ANOVA to evaluate differences in the total germination of samples following 15 days of treatments using the *anova_test* function from the *rstatix* (v 0.7.2) package (Kassambara, 2023) in R. The ANOVA related the total number of germinated seeds after 15 days of treatment as a function of the concentration of the aqueous extract. Post hoc testing was conducted using a Tukey honest significant differences test from the *rstatix* package.

### 2.2 Root sterilization experiment

An experiment was established to evaluate the influence of remnant microbial communities on washed *J. virginiana* roots used in the community root addition experiment. Seeds of *P. smithii* were germinated in autoclave‐sterilized sand. *P. smithii* was selected for use in this experiment because in previous research the species had shown sensitivity to eastern redcedar‐mediated plant–soil feedback (Bennion & Ward, 2022). When seedlings grew to ~4–6 cm in height individuals were transplanted into 2.1 L pots filled with autoclave‐sterilized Jolly Gardner C/GP® potting mix. In addition to the potting mix, roots treated using a variety of methods were added to samples. Redcedar were grown, and roots were harvested following the protocol outlined above. Roots were cut into ~3 cm lengths and thoroughly mixed. Roots were portioned into equal groups to prepare for treatment. Treatment groups included no root addition (*Control*), roots that were washed in water (*Washed*), roots that were double sterilized in an autoclave (*Auto*), roots that were treated with a combination of fungicides (*Fung*), and a 50/50 mix of the Fungicide and Auto roots (*Fung & Auto*). In the *Fungicide* treatment, 5.9 g of Captan‐50 W and 0.75 mL of Subdue Maxx were added to 1 gallon of distilled water (Domínguez‐Begines et al., 2021). Roots were submerged in the fungicide and water mixture for 10 min and then rinsed. Treatment pots had 300 mL of root material mixed evenly in the soil matrix. All plants were grown for 8 weeks under 30 W LED grow lights that were designed for seedling and vegetative growth. Following 8 weeks of growth, all samples were clipped at the root collar and aboveground biomass was collected. Biomass samples were dried in a 60°C oven for 3 days prior to being weighed. We built a linear model of *P. smithii* biomass as a function of treatment. Post hoc testing was completed using the *emmeans* (1.8.4‐1) package (Lenth, 2023) in R.

### 2.3 Grass community root addition experiment

Individuals of four common perennial grass species were grown together to evaluate if *J. virginiana* roots could generate negative legacy effects on communities that establish following the removal of *J. virginiana*. Grasses were selected to be representative of a community that co‐occurs, but also that captured the potential differences between cool‐ and warm‐season grasses. We chose *B. inermis* (smooth brome) and *E. canadensis* (Canada wildrye) as representatives of common cool‐season C_3_ grasses. Cool‐season grasses typically occur in the central mixed grass to northern shortgrass prairies of the Great Plains. *Bromus inermis* and *E. canadensis* are widespread, rhizomatous bunchgrasses of Eurasian and North American descent, respectively. We also included two warm‐season C_4_ grasses, *B. curtipendula* (side‐oats grama) and *Schizachyrium scoparium* (little bluestem); both of which are both perennial clump‐forming bunch grasses of North American origin typically occurring in warmer areas (Teeri & Stowe, 1976).

In August 2021, field soils were collected from a 60‐ to 80‐year‐old *J. virginiana* stand at the Edge of Appalachia Preserve in southern Ohio (38°42′ N, 83°26′ W). Soil samples were collected from under several large trees that were all within 100 m of each other. Large organic debris was swept away from the soil surface and samples were collected at a maximum depth of 10–15 cm. Soils were processed by running them through a 2 mm sieve and removing any rocks or organic matter. All soils were pooled and thoroughly mixed. A portion of the *J. virginiana* field soil was sterilized in an autoclave at 121°C for two 1‐h cycles (e.g. Crawford & Knight, 2017). The sterilization of soils in an autoclave can release previously unavailable nutrients into soils. We sent samples of live field soil and sterilized field soil to the Ohio State University Service Testing and Research laboratory in Wooster, OH to test for differences in soil nutrient concentrations. Live and sterilized *J. virginiana* soil was mixed at a 50:50 ratio with sterilized Jolly Gardner Pro‐line C/GP® germination mix. We added 2.4 L of live or sterilized field soil mix or germination mix to 2.8 L pots.

**FIGURE 1:**
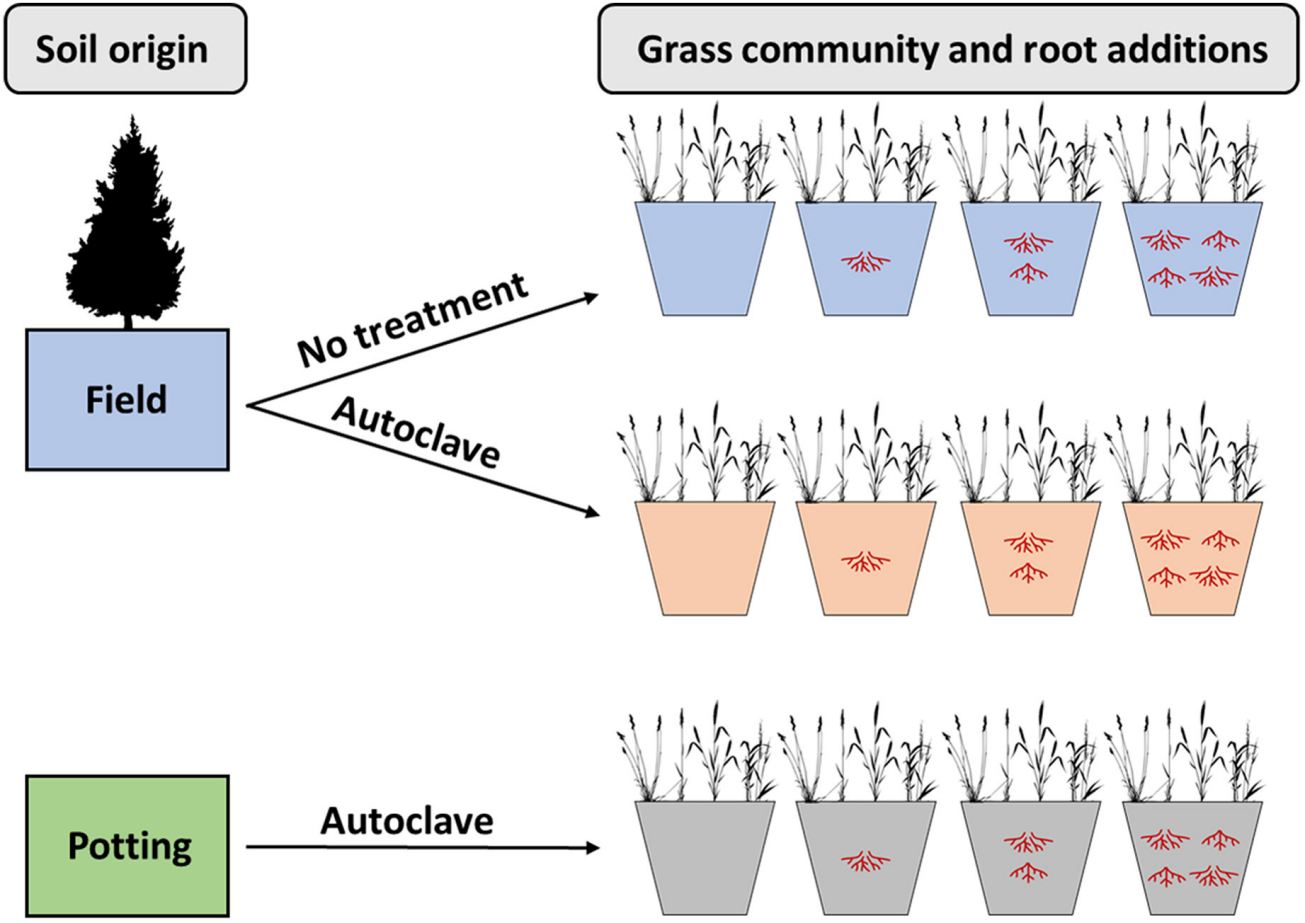
Illustration of experimental design for grass community root addition experiment. Soil origin and treatment status are indicated by color: Blue = Untreated field soil, Orange = Autoclaved field soil, Green = Untreated potting soil, and Gray = Autoclaved potting soil. The red “root structures” within pots each represent 100 mL of *Juniperus virginiana* roots. Each pot contains one individual of each of four grass species. Credit: silhouettes of plants obtained from phylopic.org.

The primary source of *J. virginiana* root material used in this experiment was obtained following the previously outlined method. A small amount of fine‐root material (<0.5 cm in diameter) collected from the field was retained for use in the experiment, these roots represented <5% of the total volume of roots used in this experiment. All roots were washed to remove any remnant soil material and then lightly processed in an industrial grinder to break them into ~2.5 cm or smaller pieces. The entire pool of roots was mixed thoroughly. Roots were added to treatment pots of each soil type at the following volumes: 0, 100, 200, or 400 mL (see Figure 1 for illustration of experimental design). Roots were mixed into the soil to be evenly distributed in each pot. There were ten replicates of each soil and root treatment combination, resulting in 120 total pots in the experiment.

In late July 2021 seeds of *B. inermis*, *E. canadensis*, *B. curtipendula*, and *S. scoparium*, seeds were germinated in preparation for the experiment. A monoculture of each species was grown in sand that had been sterilized following the procedure outlined above. One seedling of each species was transplanted into each pot in mid‐August 2021. Any individuals that died during the first week were replaced. Grasses were grown in the greenhouse for 12 weeks. Pots were watered ad libitum. Due to the experiment taking place late in the year (August–November), we used supplemental lighting from 1000 W high pressure sodium bulbs nightly from 5:00 to 8:00 pm. Grasses were harvested individually in November 2021. Aboveground biomass samples were dried in an oven at 65°C for over 48 h prior to being weighed.

### 2.4 Grass community root addition experiment statistical methods

To assess the effects of *J. virginiana* root additions on the cumulative and individual biomass of grasses, we conducted a series of analyses that calculated Hedges' *g* (standardized mean difference) effect sizes for contrasts between potting, live field soil, and sterilized field soil and levels of root addition. Hedges' *g* is an unbiased measure of the difference between two groups and is calculated by subtracting the mean of one group (*μ*_b_) from the mean of a second group (*μ*_a_) and then dividing by the pooled variance (*s*_pooled_) of the groups (Equation 1) (Hedges, 1981).

(1) $$g=\left(\mu_{a}-\mu_{b}\right)/s_{\text{pooled}}$$

We were interested in evaluating how *J. virginiana* root additions affected the biomass of grasses within the potting soil, live field soil, and sterilized field soil treatment groups. We calculated the effect of *J. virginiana* roots on cumulative shoot biomass by contrasting the treatment group with no root addition with the other three groups (100, 200, and 400 mL of roots added). For each set of contrasts the summary statistics, Hedges' *g* effect size and bias‐corrected and accelerated bootstrapped 95% confidence interval of the effect size were calculated using the *dabestr* package in R version 4.1.1 (Ho et al., 2019). We then plotted the output using a Cummings estimation plot (Ho et al., 2019). In addition to the effects of *J. virginiana* root additions on the cumulative shoot biomass of grasses in the experiment, we were interested in the response of individual species to each treatment. For each grass species, we examined the effect of each level of *J. virginiana* root addition on shoot biomass within the potting soil, live field soil, and sterilized field soil treatments. We did not make direct comparisons between the live field soil and sterile field soil, despite their shared origin. Soil testing of live field soil and sterilized field soil revealed the autoclave sterilization procedure released nutrients (notably, phosphorus) that were not available in live field soils (Table S1). For this reason, we did not consider differences between live field soil and sterilized field soil treatments derived from raw biomass as being caused solely by the soil microbial community.

In addition to evaluating the raw shoot biomass, we determined that a standardized effect ratio could be useful for comparing the effects of varying levels of root additions on the biomass of individual species between the three different soil types. Within each species and soil type combination, we calculated the log_10_ of the ratio of the biomass of grass grown with 0 mL of added root additions (Roots_0_) to the biomass of grass grown with 100, 200, or 400 mL (Roots_*x*_ where *x* is 100, 200, or 400) of roots added (Equation 2). We chose a logarithmically standardized effect ratio so the values could be compared between different soil types and between species (Crawford & Knight, 2017; see also Pernilla Brinkman et al., 2010; Petermann et al., 2008).

(2) $$\text{Effect ratio}=\log_{10}\left({\text{Roots}}_{0}/{\text{Roots}}_{x}\right)$$

The effect ratio is centered on zero when there is no effect, is positive when grass biomass is greater without roots, and is negative when the biomass of grasses grown with root additions is larger than those grown without. The data were split into potting and live and sterile field soil types. Within each soil type, we built a linear model of the effect ratio as a function of the interaction between grass species and the level of root addition. Post hoc testing was completed using the *emmeans* package in R. For each species, the estimated marginal means of the effect ratio were contrasted between each the no root addition (0 mL) and each level of root addition (100, 200, 400 mL) treatments in potting and live field soil and sterilized field soil.

Finally, we determined whether the proportion of each species' biomass to the total biomass differed among treatments. The data was split to only include field soils. For each species, we built a linear model of proportions as a function of the interaction between live or sterilized soil and the level of root addition. Post hoc testing compared the marginal means of the proportion of biomass under each treatment and for each species using the *emmeans* package in R. For each species, the estimated marginal means of proportion of shoot biomass were contrasted between the no‐root addition (0 mL) and each level of root addition (100, 200, 400 mL) treatments within live field soil and sterilized field soil types. In addition, the proportion of shoot biomass between live field soil and sterilized field soil types was contrasted at each level of root addition (0, 100, 200, and 400 mL).

## 3 RESULTS

### 3.1 Germination experiment and root sterilization experiments

**FIGURE 2:**
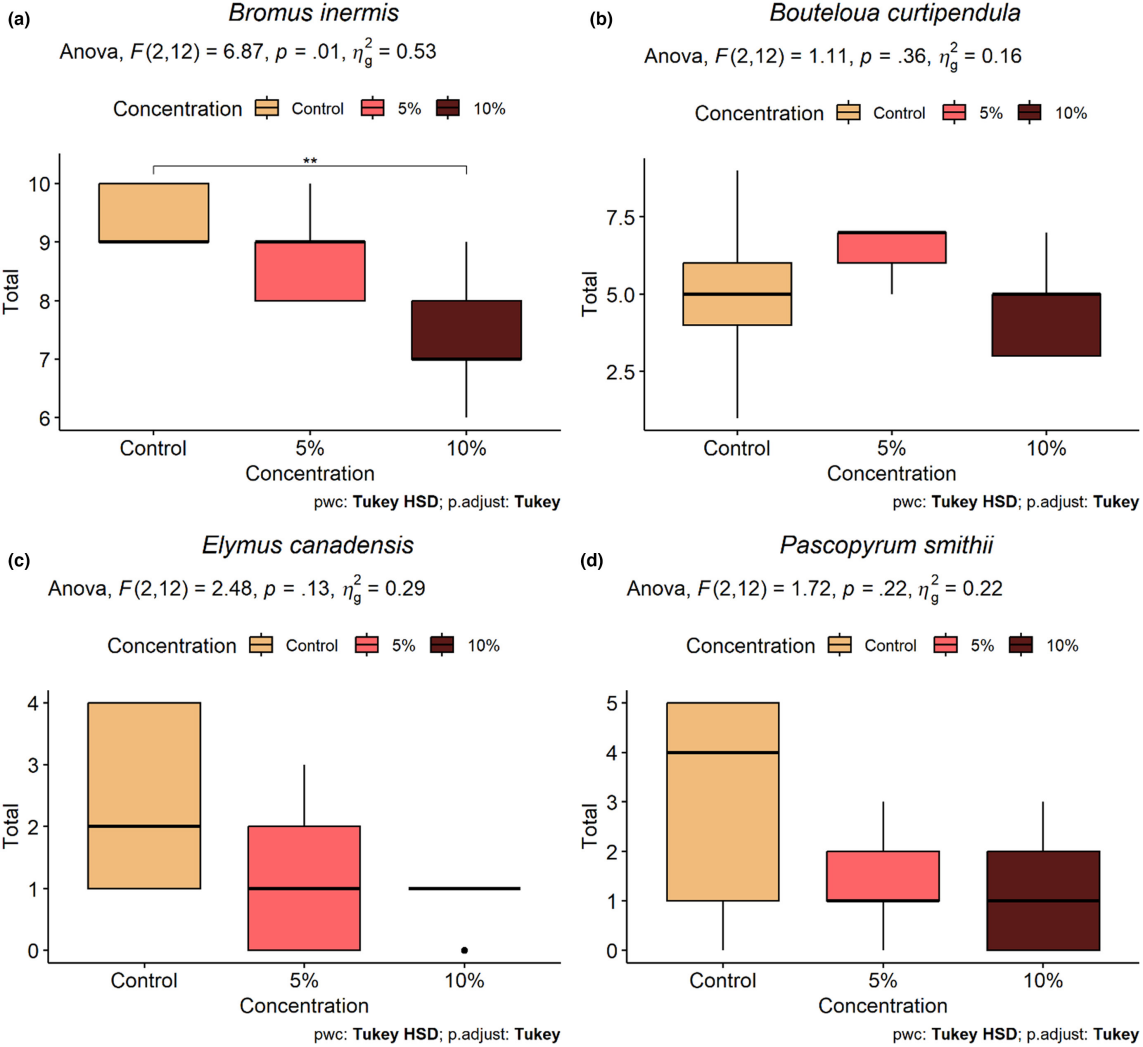
Boxplots illustrating the total number of seeds germinated (*y*‐axis) during the 15‐day experiment for each treatment. In each figure, the *x*‐axis represents the treatments control (distilled water), the 5% aqueous extract, or 10% aqueous extract concentrations. The results for (a) *Bromus inermis*, (b) *Bouteloua curtipendula*, (c) *Elymus canadensis*, and (d) *Pascopyrum smithii* are presented. Significant differences between treatment groups are denoted with asterisks.

**FIGURE 3:**
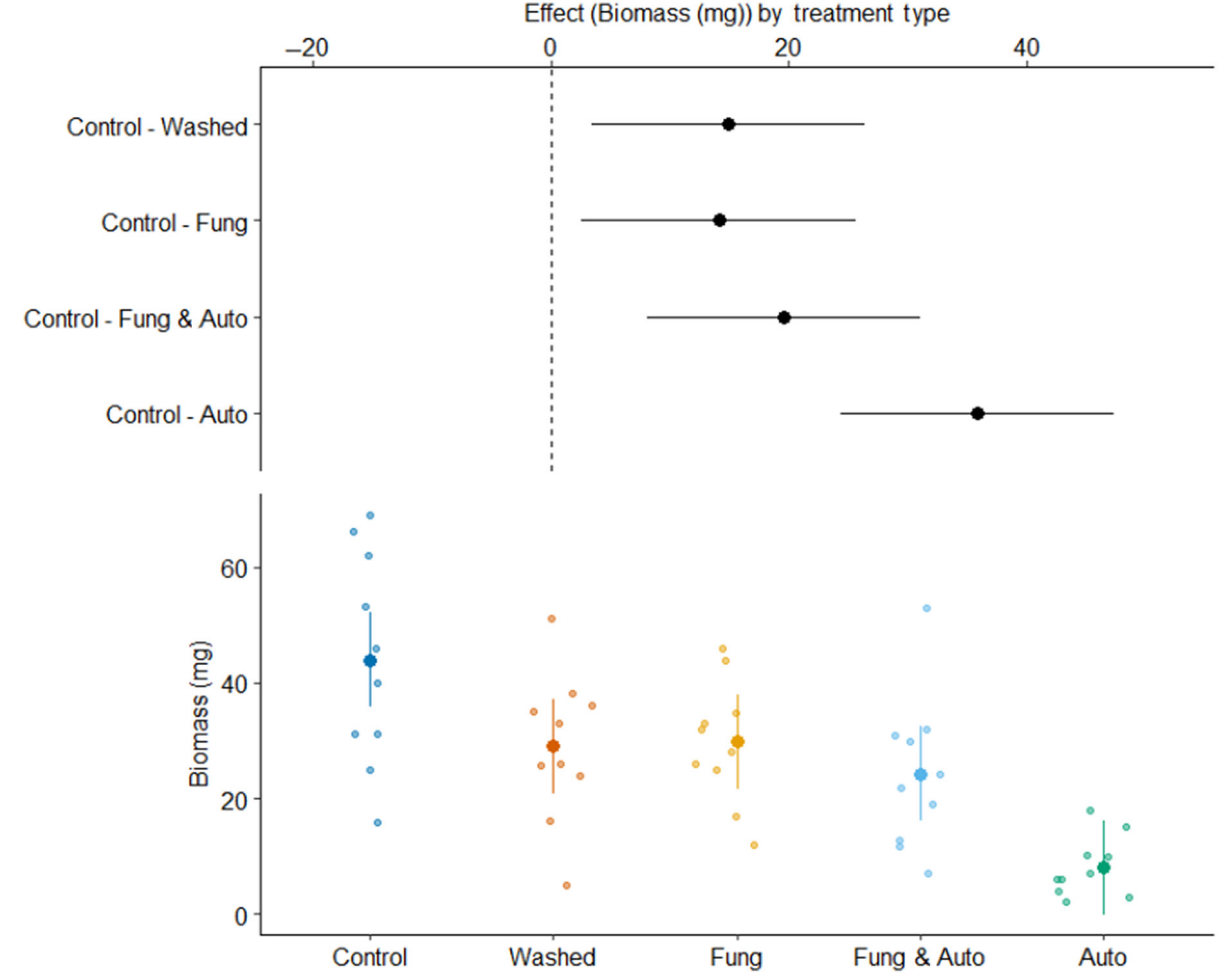
The total biomass of *Pascopyrum smithii* under various root sterilization treatments. Treatment groups include (*Control*; no roots), *Washed* (roots washed in water), *Fung* (washed roots treated with fungicide), *Autoclave* (washed roots treated in autoclave*)*, and *Fung & Auto* (a 50/50 mixture of *Fungicide* and *Autoclave* roots). The top panel in each figure shows the effects size contrasted between treatments, where dots represent the estimate and horizontal bars represent the 95% confidence intervals derived from a pooled estimate of standard deviation. The bottom panel shows the raw biomass data (dots) for each treatment. The larger dot shows the mean and vertical bar shows the standard deviation. 95% confidence intervals that do not intersect the line at *x* = 0 indicate a significant difference. Plot design from Walker (2018).

*Juniperus virginiana* root extract significantly inhibited the germination of *B. inermis* when contrasting the control and 10% concentration (*p* = .009). However, we did not detect a significant reduction in the germination rate for *B. curtipendula*, *E. canadensis*, or *P. smithii* under any root extract treatment (Figure 2). In the root‐sterilization experiment, all treatments that included *J. virginiana* roots had significantly less biomass than the control (Figure 3). Additionally, pairwise comparisons between root treatment types reveals the aboveground biomass of grasses in the autoclave treatment is significantly reduced when compared to the washed roots, fungicide, and fungicide x autoclave mixture treatments.

**FIGURE 4:**
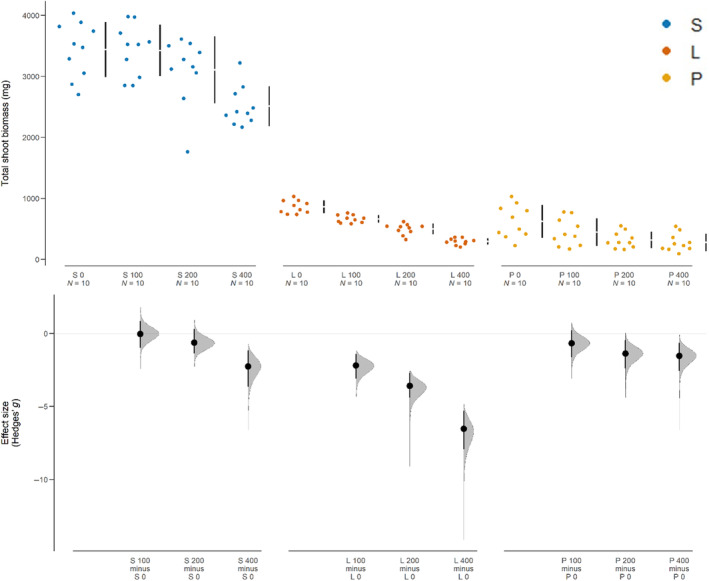
Cummings estimation plots (Ho et al., 2019) that compare the total shoot biomass of grasses grown in *Juniperus virginiana* field or potting soils with varying levels of *J. virginiana* root addition (0, 100, 200, or 400 mL). Within sterile (S), live (L), or potting (P) soils, contrasts are between the 0 mL and the 100‐, 200‐, and 400‐mL levels of root addition. The top panel shows the raw data (colored dots) and the mean and standard deviation for each group (vertical gapped lines). The bottom panel shows the Hedges' *g* effect size (black dot) and 95% confidence interval (vertical bar) for each pair of contrasts (listed on the *x*‐axis). The gray filled‐in curves represent the sampling distribution of the 95% confidence interval. 95% confidence intervals that do not intersect the line at *y* = 0 indicate a significant difference.

We evaluated the total shoot biomass of experimental community pots and found negative effects derived from additions of *J. virginiana* roots (Figure 4). In sterilized field soils, the 400 mL root‐addition treatment resulted in a substantial reduction in biomass relative to the control group. In live field soils, shoot biomass declined with each level of root addition, with the 400 mL treatment group having the largest effect. Biomass in potting soil was reduced at the 200 and 400 mL root additions.

**FIGURE 5:**
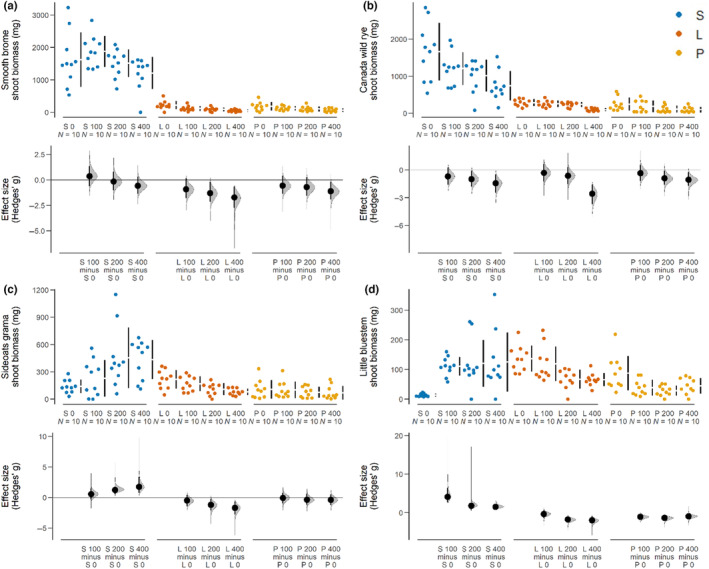
Cummings estimation plots comparing shoot biomass in control conditions with each level of *Juniperus virginiana* root addition (0, 100, 200, or 400 mL). Comparisons are grouped by soil type, either potting (P), sterile (S), or live (L), and species: (a) *Bromus inermis*, (b) *Elymus canadensis*, (c) *Bouteloua curtipendula*, or (d) *Schizachyrium scoparium*. The top panel in each plot shows the raw data (colored dots) and the mean and standard deviation for each group (vertical gapped lines). The bottom panel shows the Hedges' *g* effect size (black dot) and 95% confidence interval (vertical bar) for each pair of contrasts (listed on the *x*‐axis). The gray filled in curves represents the sampling distribution of the 95% confidence interval. 95% confidence intervals that do not intersect the line at *y* = 0 indicate a significant difference.

We assessed the raw shoot biomass of each species grouped by soil type and root addition treatment and found mixed effects (Figure 5). In live field soils, *B. inermis* shoot biomass was reduced in the 200 and 400 mL root additions. There was no detected difference in biomass between root treatments in sterile field soils for *B. inermis*. There was a reduction in biomass of *B. inermis* at the 400 mL root‐treatment level in potting soil. In live field soils, *E. canadensis* shoot biomass was reduced in the 400 mL root addition. In sterile soil, *E. canadensis* shoot biomass was reduced in the 200 and 400 mL root addition. Shoot biomass of *E. canadensis* was reduced when grown in potting soil at the 200 and 400 mL root additions. We detected increases in *B. curtipendula* biomass grown in sterile field soil at the 200 and 400 mL root additions. In contrast, *B. curtipendula* biomass grown in live field soil had a reduction in biomass at the 200 and 400 mL root additions. There was no detected difference in the biomass of *B. curtipendula* among root treatments in potting soil. Our analysis found increases in *S. scoparium* biomass grown in sterile field soil at the 100, 200, and 400 mL levels of root addition. Conversely, the biomass of *S. scoparium* was reduced in live field soil at the 200 and 400 mL root additions. In potting soil, *S. scoparium* biomass was reduced at the 100, 200, and 400 mL levels of root addition.

**FIGURE 6:**
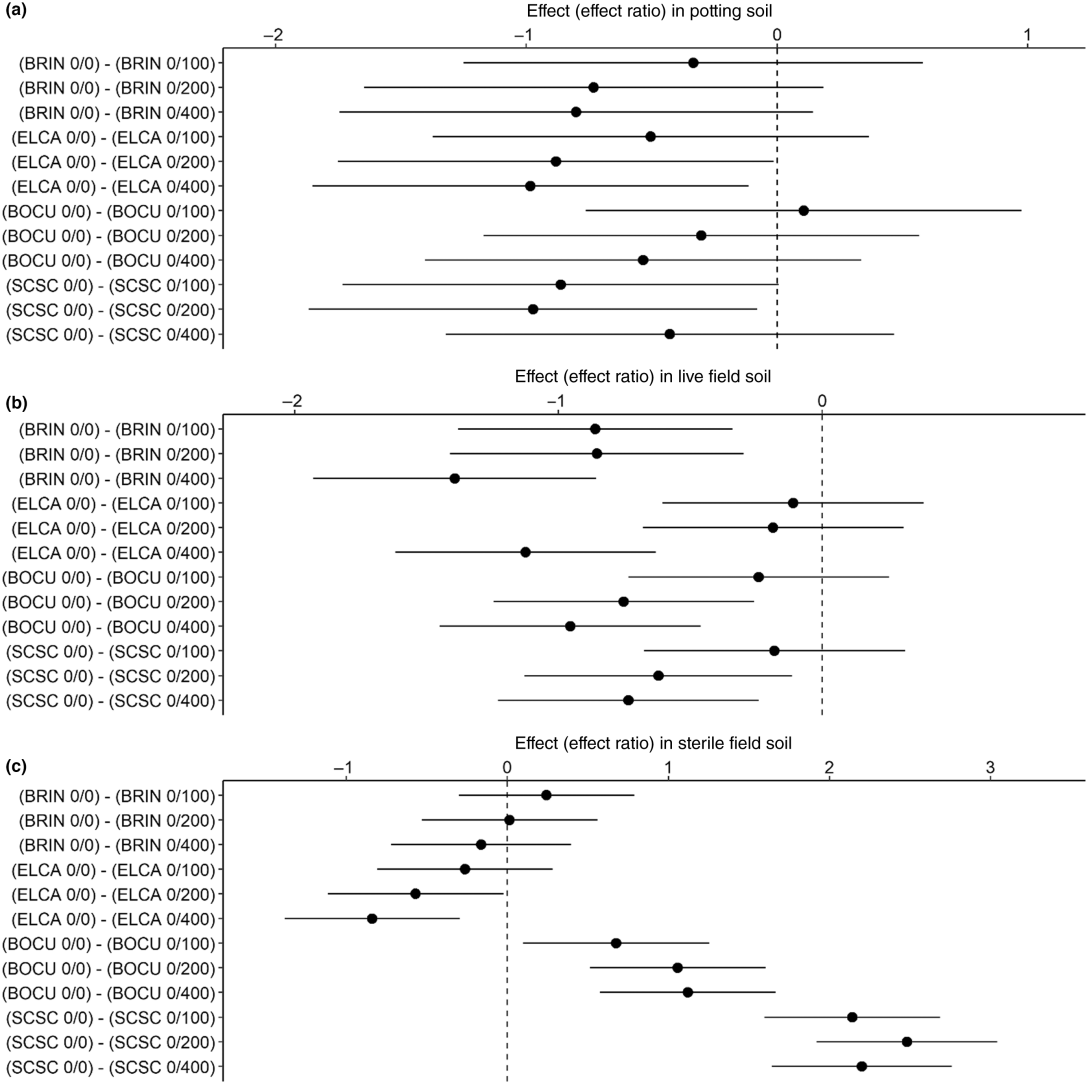
The effects plots (a–c) show the difference in the estimated marginal mean effect ratio between the control (0/0) and the three levels of root addition (0/100, 0/200, or 0/400). Dots represent the estimate and horizontal bars represent the 95% confidence intervals derived from a pooled estimate of standard deviation. In these plots (a–c), negative values indicate a reduction in biomass. All contrasts in effects plots are Bonferroni corrected. 95% confidence intervals that do not intersect the line at *y* = 0 indicate a significant difference.

We computed an effect ratio to have a standardized metric that could be used to contrast the observed effect of root treatments across the three soil types (Figure 6). In potting soil, *E. canadensis* had significant negative effect with increased root addition when comparing the control and the 200 and 400 mL effect ratios. Negative effects were observed for *S. scoparium* grown in potting soil when contrasting the control and the 200 mL effect ratios. In live field soils, *B. inermis* had strong negative effects at all root‐addition levels. *Elymus canadensis* grown in live field soil had reduced growth when contrasting the effect ratios of the control and 400 mL root‐addition treatments. Suppression of growth was observed for *B. curtipendula* when contrasting the effect ratios of the control and the 200 and 400 mL treatment groups in live soils. Similarly, *S. scoparium* had reduced growth in live soils for the contrasts of the control and the 200 and 400 mL root treatments. Sterile field soils had a mix of positive and negative effects. Contrasts between control and the 200 and 400 mL root treatments showed negative effects for *E. canadensis* growth in sterile field soils. Conversely, a net gain in biomass was shown in the contrasts of effect ratios at all levels of root treatment for *B. curtipendula* and *S. scoparium*.

**FIGURE 7:**
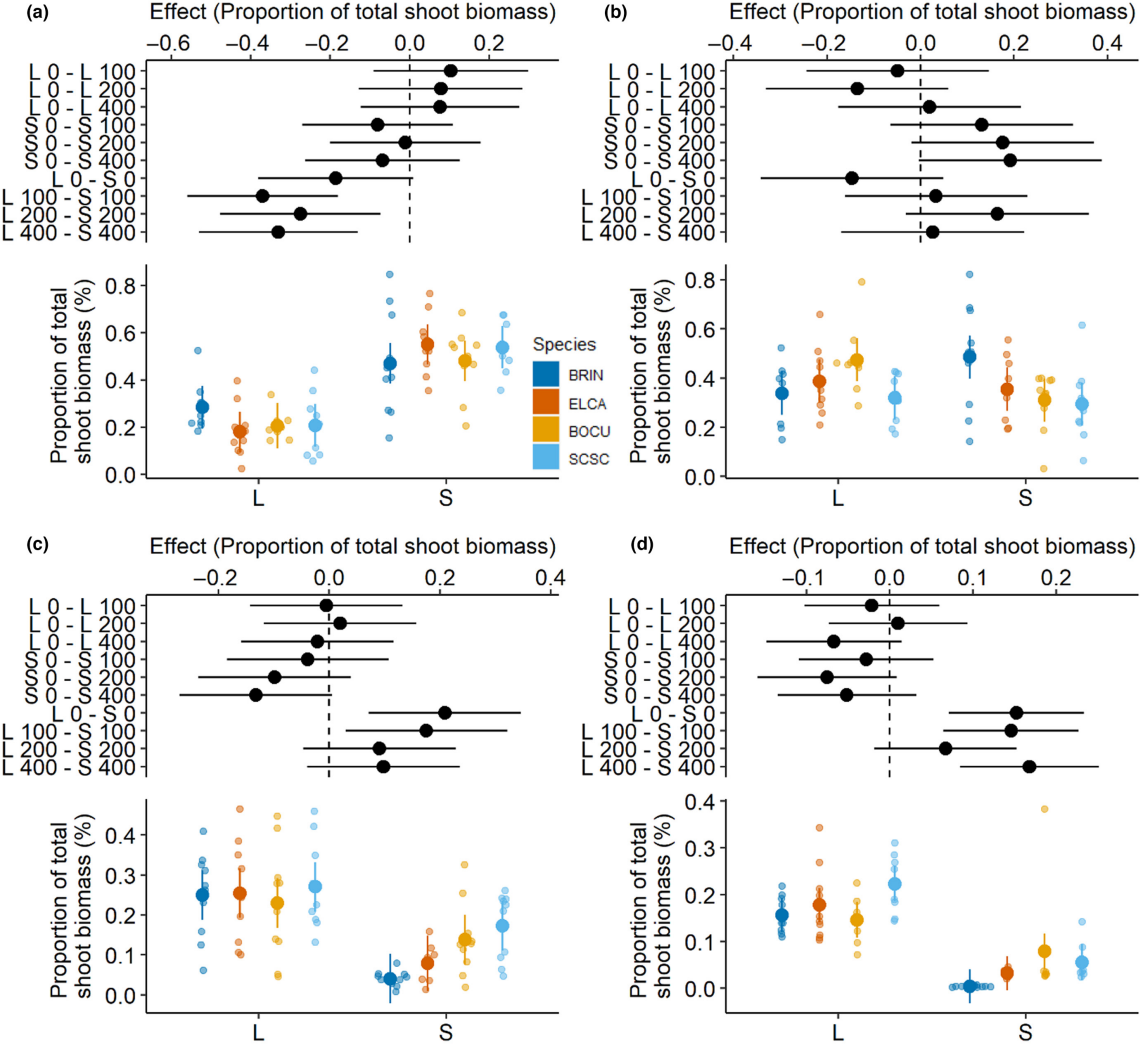
The proportion of total biomass for (a) *Bromus inermis*, (b) *Elymus canadensis*, (c) *Bouteloua curtipendula*, and (d) *Schizachyrium scoparium* in various treatments. The top panel in each figure shows the effects size contrasted between treatments within live (L) or sterile (S) soil types at each level of *Juniperus virginiana* root addition (0, 100, 200, or 400 mL). In the top panel, dots represent the estimate, and horizontal bars represent the 95% confidence intervals derived from a pooled estimate of standard deviation. The bottom panel shows the raw data (dots) for the proportion of the shoot biomass of individual species relative to the total shoot biomass in each pot. The larger dot shows the mean and vertical bar shows the standard deviation. 95% confidence intervals that do not intersect the line at *y* = 0 indicate a significant difference. Plot design from Walker (2018).

Within each soil type, we evaluated the overall proportion of biomass of each species relative to the total biomass in each pot at each level of root addition treatment. The proportion of *B. inermis* biomass did not change under root treatments in live or sterile field soil. However, when comparing live and field soils, the proportion of *B. inermis* biomass was reduced in the 0 mL (*t* ratio = −3.15, *p* = .047), 100 mL (*t* ratio = −5.44, *p* < .001), 200 mL (*t* ratio = −4.64, *p* < .001), and 400 mL (*t* ratio = −4.37, *p* = .001) levels of root treatment (Figure 7a). No difference in proportion of biomass was detected for *E. canadensis* at each level of root treatment within or between live or sterile field soil types (Figure 7b). Within live and sterile field soils, the proportion of *B. curtipendula* biomass did not differ at any level of root treatment. However, the proportion of *B. curtipendula* biomass increased in live compared to sterilized soil at the 0 mL (*t* ratio = 4.74, *p* < .001) and 100 mL (*t* ratio = 3.76, *p* < .01) root‐treatment levels (Figure 7c). No difference was observed in the proportion of *S. scoparium* within live or sterile soils at each level of root treatment. We found an increase in the proportion of *S. scoparium* biomass from the live soil group when compared to sterile soils at the 0 mL (*t* ratio = 5.68, *p* < .001), 100 mL (*t* ratio = 5.43, *p* < .001), and 400 mL (*t* ratio = 6.46, *p* < .001) levels of root treatment (Figure 7d).

## 4 DISCUSSION

The conversion of grasslands in the United States to *J. virginiana* woodlands has slowed in areas with active management but is generally increasing across its range (Filippelli et al., 2020; Miller et al., 2017). We found that *J. virginiana* root material inhibited the germination of certain species and reduced the overall productivity of an experimental grass community. In this experiment, we added root biomass to three different soil types and observed an overall reduction in biomass with increasing root addition. It should be noted that roots were not sterilized prior to use in this experiment. They were washed thoroughly, but small amount of soil microbes likely remained and were transferred into experimental pots.

The root sterilization experiment indicates that the transfer of soil microbes could not be solely responsible for the suppression of growth observed in the community root addition experiment. In the root‐sterilization experiment, there was no difference in the biomass production of live, washed *J. virginiana* roots, and roots that had been treated with a fungicide. However, the autoclave root treatment significantly reduced biomass production relative to all other root treatments. This effect could be from the removal of plant‐growth promoting bacteria on the surface of the roots which further reduces plant performance (Glick, 2012) or due to the heat and pressure of the autoclave changing the release rate of chemicals from the roots (e.g. Choi et al., 2020). In the case of *J. virginiana*, we suspect secondary metabolites are being released from roots into surrounding soil that inhibit the growth of grasses. Secondary metabolites exuded into soils from roots or litter can have allelopathic effects that range in effect from dampening positive microbial feedbacks to strongly suppressing the growth of certain species (Bennett & Klironomos, 2019). Allelochemicals disrupt the function of plants by inhibiting their associated fungal communities or through direct phytotoxicity (Inderjit et al., 2011). The allelopathic effect of secondary metabolites may only exist when they are actively being exuded into the soils or can persist long after the source has been removed (Del Fabbro & Prati, 2015). In addition to the allelopathic effect of *J. virginiana* roots, the soil microbial community near *J. virginiana* has been documented to have a negative feedback on grasses (Bennion & Ward, 2022). The inhibition of growth in prairie grass communities by *J. virginiana* soils and roots could make formerly occupied sites more susceptible to reestablishing *J. virginiana*.

We propose *J. virginiana* roots could contribute to negative ecological legacy effects that promotes the maintenance of *J. virginiana*‐dominated system and that these effects could persist after *J. virginiana* death. The phenomenon of organic materials such as the roots or litter of invasive species inducing hysteresis in systems for years following treatment has been well documented (Grove et al., 2012; Nsikani et al., 2018; Reynolds et al., 2017). Recently, more researchers have begun examining the potential for native woody encroachers to create legacy effects while in situ and following removal (Eldridge & Ding, 2021). For example, microbially mediated plant–soil feedback generated by a range‐expanding shrub has been found to inhibit the performance of conspecific seedlings (Collins et al., 2023). If the effect we observed experimentally exists in field conditions, then legacy effects of *J. virginiana* roots could be considered a mechanism that favors the reestablishment of the species in treated areas. Furthermore, the strength of the suppressive effect increased at higher levels of root addition. In field conditions, *J. virginiana* roots are fibrous and widespread near the surface of soils (Burns, 1990; Hiziroglu & Zhang, 2010), and exist in the rooting zone of newly establishing grasses (Hamati, 2022). Therefore, areas with a particularly high concentration of *J. virginiana* roots may inhibit grass growth enough to provide pockets of refugia suitable for *J. virginiana* establishment. *J. virginiana* root‐laden soils are unlikely to eliminate grass establishment, but their suppressive strength may provide vegetation gaps to favor their return.

The outcomes of recent case studies on *J. virginiana* treatments align with our proposed mechanism of legacy effects derived from *J. virginiana* roots (e.g., Bielski et al., 2021; Fogarty et al., 2021). For example, Bielski et al. (2021) found the use of intense prescribed fire can result in high mortality of *J. virginiana* and the temporary restoration of a diverse and productive grassland community. These outcomes are short lived, with *J. virginiana* reestablishing within 1–2 years following treatment (Fogarty et al., 2021). In the absence of seed limitation, we suggest the potential legacy effect we observed in this experiment could represent a key factor in the rate of reestablishment. The observed window for *J. virginiana* reestablishing following treatment according to Fogarty et al. (2021) is short enough (1–2 years) that even fine *J. virginiana* root materials should remain in the soil matrix. This means that the allelopathic effect of *J. virginiana* roots will likely persist long enough for seedlings to establish. It should be noted that there is no evidence of self‐suppression for *J. virginiana* when growing in soil previously occupied by *J. virginiana* (Bennion & Ward, unpublished data).

Our findings indicate the impact of *J. virginiana* on the plant community persists following its removal. The legacy effect of *J. virginiana* is an example of a mechanism of encroachment that is not readily apparent but may have a significant impact on the success of the species. Further studies could evaluate the strength of this mechanism under field conditions through experimental removal of *J. virginiana* and monitoring the growth of establishing species.

## AUTHOR CONTRIBUTIONS

**Leland D. Bennion:** Conceptualization (lead); data curation (lead); formal analysis (lead); visualization (lead); writing – original draft (lead); writing – review and editing (equal). **David Ward:** Funding acquisition (lead); supervision (lead); writing – review and editing (equal).

### OPEN RESEARCH BADGES

This article has earned an Open Data badge for making publicly available the digitally‐shareable data necessary to reproduce the reported results. The data is available at [https://doi.org/10.21038/benn.2023.1001].

## Supporting information


Table S1.

## Data Availability

Our data are archived in the Open Access Kent State (OAKS) repository. They can be viewed at: https://doi.org/10.21038/benn.2023.1001.
