# Supplementary material for: Eastern redcedar roots create legacy effects that suppresses growth of prairie species
Source: Ecol Evol. 2023 Dec 11;13(12):e10775. doi: 10.1002/ece3.10775 (PMC10711520; doi:10.1002/ece3.10775)
Supplement: Supplementary file 1 — Table S1. [file ECE3-13-e10775-s001.docx]

Supplement

Table S1. The results of a soil analysis on three samples of live or sterilized field soils. Note the difference in phosphorus (P) between live and sterilized soils.

|  |  |  | **Bray P-1** | **Ammonium Acetate Extract** | | |  |  |  |  |  |  |  |
| --- | --- | --- | --- | --- | --- | --- | --- | --- | --- | --- | --- | --- | --- |
|  |  |  | **µg/g** | **µg/g** | **µg/g** | **µg/g** | **meq/ 100g** | **Base Saturation** | | | **OM** | **N** | **C** |
| **ID** | **pH** | **LTI** | **P** | **K** | **Ca** | **Mg** | **CEC** | **% Ca** | **% Mg** | **% K** | **%** | **%** | **%** |
| Live 1 | 7.0 | 70.0 | 4.99 | 219 | 3406 | 650.4 | 23.0 | 74.0 | 23.6 | 2.4 | 10.5 | 0.37 | 5.13 |
| Live 2 | 6.9 | 70.0 | 4.64 | 210 | 3283 | 623.9 | 22.2 | 74.1 | 23.5 | 2.4 | 10.4 | 0.38 | 5.03 |
| Live 3 | 6.9 | 70.0 | 4.69 | 213 | 3419 | 647.9 | 23.0 | 74.2 | 23.4 | 2.4 | 10.6 | 0.38 | 5.16 |
| Sterile 1 | 6.6 | 69.2 | 14.18 | 202 | 3395 | 542.2 | 23.0 | 73.9 | 19.7 | 2.3 | 10.2 | 0.38 | 5.08 |
| Sterile 2 | 6.6 | 69.7 | 13.41 | 204 | 3344 | 549.8 | 22.2 | 75.4 | 20.7 | 2.4 | 9.7 | 0.39 | 5.07 |
| Sterile 3 | 6.6 | 68.9 | 13.83 | 200 | 3375 | 546.0 | 23.3 | 72.6 | 19.6 | 2.2 | 10.2 | 0.38 | 5.01 |
